# Skeletal muscle mitochondrial marker responses to a single bout and 6 weeks of high load versus high volume resistance training in previously trained men

**DOI:** 10.1113/EP093172

**Published:** 2026-03-24

**Authors:** Breanna Mueller, Carlton D. Fox, Hailey A. Parry, Paulo H. C. Mesquita, Christopher G. Vann, Bradley A. Ruple, Casey L. Sexton, Joshua S. Godwin, Mason M. McIntosh, Darren T. Beck, Kaelin C. Young, Stuart M. Phillips, Andreas N. Kavazis, Michael D. Roberts

**Affiliations:** ^1^ School of Kinesiology Auburn University Auburn Alabama USA; ^2^ Department of Cell Biology and Physiology Edward Via College of Osteopathic Medicine ‐ Auburn Campus Auburn Alabama USA; ^3^ Biomedical Sciences Pacific Northwest University of Health Sciences Yakima Washington USA; ^4^ Department of Kinesiology McMaster University Hamilton Ontario Canada

**Keywords:** high load resistance training, high volume resistance training, mitochondria, skeletal muscle, transcriptional response, translational response

## Abstract

The effects of high‐load (HL) versus high‐volume (HV) resistance training (RT) on various molecular outcomes are similar. However, mitochondrial responses remain understudied. Therefore, the purpose of this study was to interrogate mitochondrial mRNA and protein responses to acute and chronic HL versus HV RT. Vastus lateralis biopsies from resistance trained males in two prior studies were assessed. In Study 1, 11 college‐aged men completed an acute bout of either HL or HV RT exercises to failure. Biopsies were collected at PRE, 3‐h post‐, and 6‐h post‐exercise. In Study 2, 15 college‐aged men participated in 6 weeks of supervised unilateral RT where each leg was assigned to either HL or HV RT. Biopsies were collected from both legs prior to and 72 h following last training bout of the intervention. Biopsies from both studies were used to assess mitochondrial mRNAs, and Study 2 biopsies were assayed for mitochondrial proteins and citrate synthase (CS) activity. Results from both studies revealed several significant main effects of time but no significant interactions. Additionally, CS activity, a surrogate of mitochondrial content, decreased following chronic RT (*P* = 0.016) but no interaction was observed between the HV and the HL leg over time (*P* = 0.882). In conclusion, while RT resulted in both acute mitochondrial mRNA and chronic CS activity and mitochondrial protein responses, there were no differences in the HL versus HV paradigms on these outcomes.

## Abstract (highlights)

- **What is the central question of this study?**
  Whether different styles of resistance training promote different muscle mitochondrial adaptations.
- **What is the main finding and its importance?**
  The data provide continued support that resistance training promotes mitochondrial adaptations. However, training load acutely or over 6 weeks does not primarily influence mitochondrial adaptations so long as training effort is performed in close proximity to volitional fatigue.

## 1 INTRODUCTION

Skeletal muscle is a highly plastic tissue that responds to applied stimuli (Adams & Bamman, 2012; Bodine, 2013; Egan & Sharples, 2023). Perhaps the most well‐studied of these adaptations is hypertrophy in response to some external form of mechanical overload (Roberts et al., 2023). Resistance training (RT) is the most readily available form of mechanical overload for humans, and as such, many studies have aimed at optimizing skeletal muscle hypertrophy via modifications to RT protocols. One prominent modification is the alteration of load (e.g. weight lifted) or volume (e.g. number of sets or repetitions performed) (Schoenfeld et al., 2017). As such, studies examining acute responses or training adaptations to high volume (HV) versus high load (HL) RT have gained widespread interest (Burd et al., 2010; Davies et al., 2022; Grisebach et al., 2024; Mitchell et al., 2012; Morton et al., 2016, 2019; Nitzsche et al., 2020; Sexton et al., 2023; Vann et al., 2022). HL RT typically involves lifting heavier weight for fewer repetitions (e.g. 3–5 sets at 80% or more of one repetition maximum (1RM) for 3–5 repetitions), while HV RT involves lifting lighter weight while performing more repetitions per set comparatively (e.g. sets containing >10 repetitions per set with <65% of 1RM) (Kraemer & Ratamess, 2004). Though HL training accomplishes greater increases in 1RM relative to HV training, aggregate data suggest that both HL and HV training to failure result in similar hypertrophic adaptations (Carvalho et al., 2022; Schoenfeld et al., 2017).

Although strength and hypertrophy adaptations between HL and HV training have been compared, little evidence to date exists regarding muscle mitochondrial adaptations that occur with each form of training. Lim et al. (2019) recruited college‐aged males to perform 10 weeks of either 80FAIL training (80% 1RM to volitional fatigue), 30WM training (30% 1RM volume matched to 80FAIL) or 30FAIL training (30% 1RM to volitional fatigue) RT where pre‐ and post‐intervention biopsies were examined for mitochondrial proteins indicative of mitochondrial volume, mitochondrial biogenesis and remodelling. While these authors reported an increase in a marker of mitochondrial capacity (COX IV protein expression increased after training but no group–time interaction was observed), there were no changes in markers of mitochondrial biogenesis. The authors reported 30WM and 30FAIL training increased markers of mitochondrial biogenesis (peroxisome proliferator‐activated receptor γ coactivator 1‐α (PGC1α), transcription factor A, mitochondrial (TFAM), and nuclear respiratory factor 1 (NRF1)) and remodelling (mitofusin 2 (MFN2), optic atrophy 1 (OPA1), dynamin‐related protein 1 (DRP1), PTEN‐induced kinase 1 (PINK1) and Parkin) compared to 80FAIL training. This is perhaps indicative of differential demands in energy production and mitochondrial activity between HL versus HV RT. Indeed, it has been suggested that HV RT might contribute to greater impacts to the mitochondria than HL RT (Parry et al., 2020).

We recently published a study involving previously trained men who performed a single bout of HL and HV training, and transcriptomic data were analysed using bioinformatics. This study is referred to as Study 1 herein (Sexton et al., 2023). While these data were informative, genes related to mitochondrial biogenesis and remodelling were not highlighted with bioinformatics, nor were they manually interrogated. Likewise, we recently published a separate study in another cohort of previously trained men who performed a 6‐week unilateral leg RT programme (3 days/week), where one leg performed a HV paradigm and the other performed a HL paradigm (Vann et al., 2022). This study is referred to as Study 2 herein. Interestingly, the HV leg presented an elevation at 6 weeks of integrated non‐myofibrillar protein synthesis rates versus the HL leg. This finding may have been related to the expansion of non‐contractile proteins in the HV versus HL leg (e.g., metabolic or mitochondrial proteins). However, again, mitochondrial markers and citrate synthase (CS) activity were not assessed in our original investigation. Given that mitochondrial responses to HV or HL RT have been less investigated relative to other molecular markers (e.g., MyoPS, ribosomes, satellite cells), we leveraged specimens from the aforementioned studies to interrogate how HL and HV training affected markers of mitochondrial biogenesis and remodelling. Note, the genes and proteins we interrogated in this secondary analysis were chosen based on our examining these in other RT studies performed by our laboratories (Mesquita et al., 2020, 2023) as well as being similar targets examined by Lim and colleagues (Lim et al., 2019). Based on the limited research presented above, we hypothesized HV training would increase markers of mitochondrial biogenesis and remodelling as well as CS activity relative to HL training.

## 2 METHODS

### 2.1 Ethical approval

All procedures were approved by the Institutional Review Board at Auburn University (Protocols #20‐081 MR 2003 and #19‐245 MR 1907), and the studies described below conformed to the standards set forth by the latest revision of the *Declaration of Helsinki* except for not being registered as clinical trials. The methods outlined below are a partial report of relevant methods and procedures performed in Study 1 (Sexton et al., 2023) and Study 2 (Vann et al., 2022). A comprehensive description of the original data collections comprising this investigation can be found in Sexton et al. (2023) and Vann et al. (2022).

### 2.2 Participant inclusion criteria

Participants were recruited verbally and through fliers posted at Auburn University. Eligible participants from both studies: (i) self‐reported ≥12 months of RT, (ii) were free of cardiometabolic diseases (e.g., type II diabetes, severe hypertension), and (iii) did not possess conditions precluding participation in the RT or the collection of skeletal muscle biopsies. Interested participants provided verbal and written informed consent to participate in the studies before the data collection procedures outlined below. Eleven male participants were included in the Study 1 analysis, and 15 separate participants were included in the Study 2 analysis unless stated otherwise.

### 2.3 Study designs and biochemical assays

**FIGURE 1:**
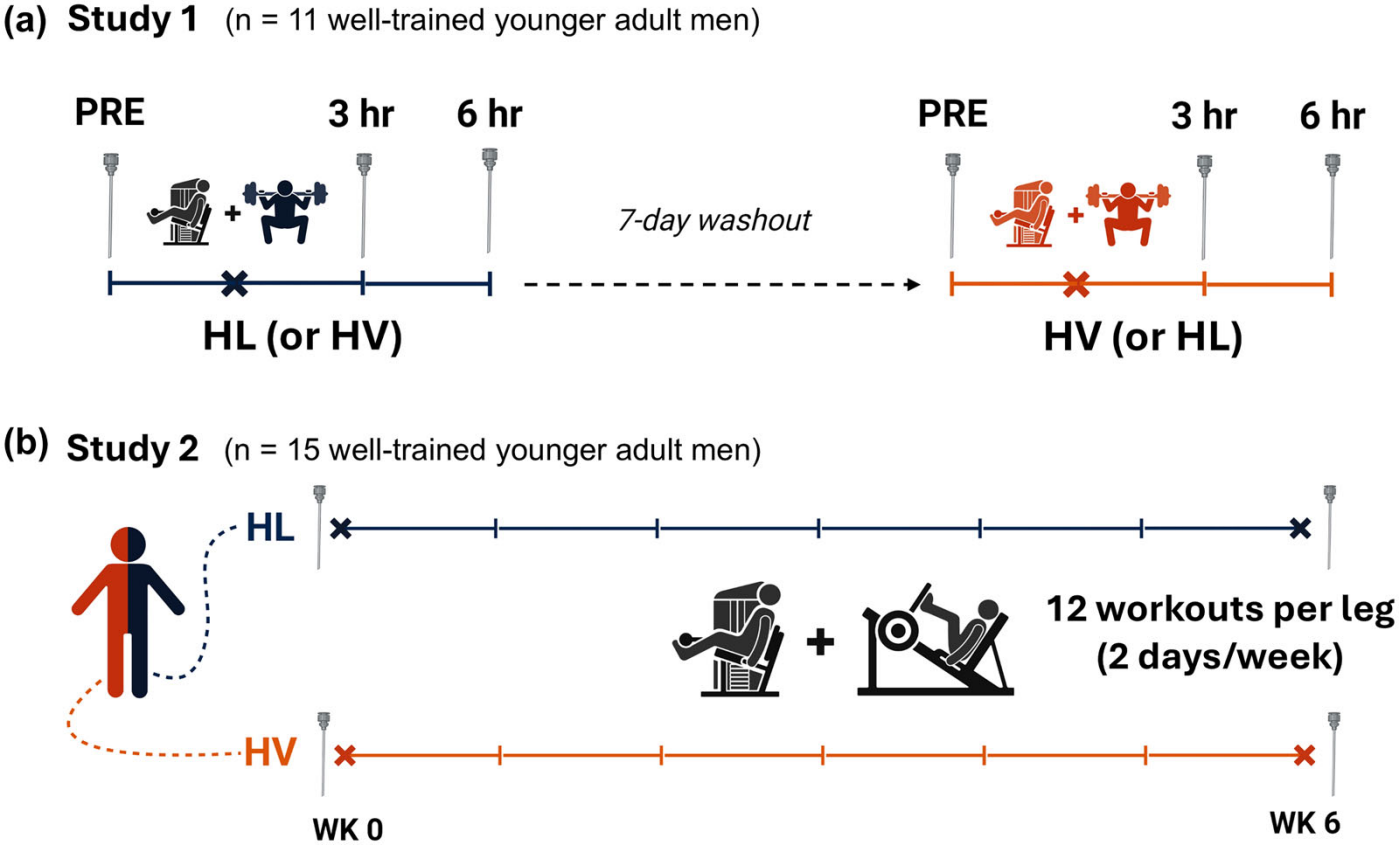
Study designs. (a) Study 1: participants performed a single bout of high volume (HV) resistance exercise (RE) or a single bout of high load (HL) RE in a randomized, crossover design with a 7‐day washout period between testing sessions. Skeletal muscle biopsies were taken prior to as well as 3 and 6 h after each exercise bout. (b) Study 2: participants performed 6 weeks of unilateral RT where the left and right legs of each participant were randomly assigned to perform either HV or HL resistance exercise. Skeletal muscle biopsies were taken at PRE and POST of the training intervention. Needle symbol represents a biopsy. HL, high load; HV, high volume; WK, week. Created using BioRender.

Figure 1a is a schematic illustration of Study 1. In this study, participants made 3 visits to the laboratory. The first visit included a vastus lateralis muscle biopsy (PRE) and back squat and leg extension one repetition maximum (1RM) test. These 1RM values served to prescribe training loads for each of the following two visits during which participants performed four sets of back squats and leg extensions to failure using either 30% 1RM (HV) or 80% 1RM (HL) in a randomized, within‐subject design. Vastus lateralis muscle biopsies were collected prior to as well as 3‐ and 6‐h post‐exercise, and all time points were processed for transcriptomic analysis as described by Sexton et al. (2023). Figure 1b is a schematic illustration of Study 2. In this study, participants completed 6 weeks of total body RT (3×/week). Prior to the start of training, baseline vastus lateralis muscle biopsies were collected (PRE) and 1RM values for relevant RT exercises were determined. Lower body exercises included unilateral leg press and unilateral leg extension to allow for contrasting training schemes to be carried out within‐subject. Left and right legs were randomly assigned to either HV or HL unilateral training. The HV leg began training performing sets of 10 repetitions at 60% 1RM and increased training volume over 6 weeks. The HL leg began training performing sets of five repetitions at 80% 1RM and increased training load over 6 weeks. Vastus lateralis muscle biopsies were collected at the conclusion of the training intervention (POST) 72 h following the last supervised workout. Notably, participants also performed three sets of 10‐repetition barbell bench press, pronated grip barbell row, and barbell stiff‐leg deadlift exercises during the 3 days per week training sessions to minimize RT outside of the study. Although outside physical activity levels were not monitored, participants were instructed to generally maintain their pre‐intervention physical activity levels while strictly avoiding any forms of resistance or cardiovascular exercise training outside of the study.

#### 2.3.1 Data mining for mRNAs of interest from Study 1

Genes investigated in Study 1 were selected from a transcriptome‐wide mRNA analysis that was previously attained and described by Sexton et al. (2023). All 11 participants possessed PRE and 3‐h post‐exercise values, whereas only 10/11 participants possessed enough tissue to analyse 6‐h post‐exercise values. Processing involved obtaining ∼10 mg of skeletal muscle from −80°C and placing tissue on a liquid nitrogen‐cooled ceramic mortar. Tissue was homogenized in Trizol (VWR; Radnor, PA, USA) to isolate RNA as described by Sexton et al. (2023). Next, purity was inspected using a NanoDrop Lite Spectrophotometer (Thermo Fisher Scientific, Waltham, MA, USA). Finally, samples were shipped on dry ice to a commercial laboratory for transcriptome‐wide analysis using the Clariom S Assay Human mRNA array (North American Genomics; Decatur, GA, USA) and the raw data .CEL file received was analysed using the Transcriptome Analysis Console v4.0.2 (Thermo Fisher Scientific). mRNAs involving mitochondrial biogenesis (PGC1α, TFAM, NRF1), fusion (MFN2, OPA1), fission (DRP1) and mitophagy (Pink1, Parkin) were individually selected from this data set and subjected to statistical analysis using mixed effects models to account for the 6‐h post‐exercise missing time point.

#### 2.3.2 Data mining for mRNAs of interest from Study 2

RNA was isolated from a subset of participants (12 of the 15 participants) whereby ∼10 mg skeletal muscle tissue was homogenized using Trizol (VWR) and analysed using the Human Transcriptome Array 2.0 (Affymetrix). Array results were analysed using the Gene Titan system (Thermo Fisher Scientific). Raw .CEL files were read into R (v4.1) and data were normalized using robust multi‐array normalization (Irizarry et al., 2003), and probes were annotated using the BioConductor package ‘Affy’ and the HTA2.0 library. Probes for the present analysis were extracted and analysed via two‐way repeated measures ANOVA, described further below.

#### 2.3.3 Western blotting for Study 2

Protein expression data for all 15 Study 2 participants were attained via western blot analysis as previously detailed by Vann et al. (2022). Briefly, lysates obtained were prepared for western blotting using 4× Laemmli buffer at 1 µg/µL. Following sample preparation, 15 µL samples were loaded onto pre‐casted gradient (4–15%) SDS‐polyacrylamide gels (Bio‐Rad Laboratories, Hercules, CA, USA) and subjected to electrophoresis (180 V for 45–60 min) using pre‐made 1× SDS‐PAGE running buffer (VWR). Proteins were subsequently transferred (200 mA for 2 h) to polyvinylidene difluoride membranes (PVDF) (Bio‐Rad), Ponceau stained, and imaged to ensure equal protein loading between lanes. Membranes were then blocked for 1 h at room temperature with 5% non‐fat milk powder in Tris‐buffered saline with 0.1% Tween‐20 (VWR). Rabbit anti‐PGC1α (1:1000; cat. no.: GTX37356; GeneTex, Irvine, CA, USA), rabbit anti‐NRF1 (1:2000, cat. no. GTX103179, 11168915, GeneTex), rabbit anti‐TFAM (1:2000, cat. no. H00007019‐D01P,171 5621, Abnova Corporation, Taoyuan City, Taiwan), rabbit anti‐MFN2 (1:2000, cat. no. 3882‐100; BioVision, Milpitas, CA, USA), rabbit anti‐DRP1 (1:2000, cat. no. NB110‐55288SS; Novus Biologicals USA, Littleton, CO, USA), rabbit anti‐Parkin (1:2000, cat. no. 2132, 10693040, Cell Signaling Technology, Danvers, MA, USA), rabbit anti‐PINK1 (1:2000, cat. no. 6946, 11179069, Cell Signaling Technology) were incubated with membranes overnight at 4°C in TBST with 5% bovine serum albumin (BSA). The following day, membranes were incubated with horseradish peroxidase (HRP)‐conjugated anti‐rabbit IgG (cat. no.: 7074; Cell Signaling Technology) in TBST with 5% BSA at room temperature for 1 h (secondary antibodies diluted 1:2000). Membrane development was performed using an enhanced chemiluminescent reagent (Luminata Forte HRP substrate; EMD Millipore, Billerica, MA, USA), and band densitometry was performed using a gel documentation system and associated software (Bio‐Rad). Raw densitometry values for each target were divided by whole‐lane Ponceau densities, and these data were statistically analysed between groups.

#### 2.3.4 Citrate synthesis activity assay for Study 2

Muscle tissues stored in foil from all Study 2 participants (*n* = 15) were removed from −80°C and placed on a liquid nitrogen‐cooled ceramic mortar. Crushed tissue (∼20 mg) was weighed on a laboratory scale exhibiting a sensitivity of 0.1 mg (Mettler‐Toledo; Columbus, OH, USA), and tissue was quickly placed in 1.7 mL tubes containing 200 µL lysis buffer (25 mM Tris, pH 7.2, 0.5% Triton X‐100, 1× protease inhibitors). Samples were homogenized using tight‐fitting hard‐plastic microtube pestles on ice and centrifuged at 1500 *g* for 10 min at 4°C. Supernatants were collected and placed in new 1.7 mL microtubes on ice for protein determination. Protein concentrations were determined the same day using a commercially available BCA kit (Thermo Fisher Scientific). Samples were assayed in duplicate using a microplate assay protocol (20 µL of 5× diluted sample + 200 µL Reagent A + B). Samples were then stored at −80°C until assays described below.

Tissue lysates obtained above were batch‐processed for CS activity as previously described by our laboratory (Ruple et al., 2021). This metric was used as a surrogate for mitochondrial content per the findings of Larsen et al. (2012), suggesting CS activity correlates with transmission electron micrograph images of mitochondrial content (*r* = 0.84, *P* < 0.001). The assay principle is based upon the reduction of 5,50‐dithiobis (2‐ nitrobenzoic acid) (DTNB) at 412 nm (extinction coefficient 13.6 mmol/L/cm) coupled to the reduction of acetyl‐CoA by the CS reaction in the presence of oxaloacetate. Skeletal muscle protein (5 µg) from each biopsy was assayed in duplicate where samples were added to a mixture composed of 0.125 mol/L Tris–HCl (pH 8.0), 0.03 mmol/L acetyl‐CoA, and 0.1 mmol/L DTNB. The reaction was initiated by adding 5 µL of 50 mmol/L oxaloacetate and the absorbance change was recorded for 1 min. The coefficient of variation for all duplicates was 6.1%.

### 2.4 Statistical analysis

Data collected from both studies were statistically analysed and graphed using GraphPad Prism v10.2.2 (GraphPad Software, Boston, MA, USA). Normality was assessed using the Shapiro–Wilk test and non‐normal data (TFAM protein) were log transformed. Two‐way, repeated measures ANOVA (or mixed effect analysis where a participant datapoint was missing) was used to determine the interaction between and/or main effects of time (Study 1: PRE, 3 and 6 h; Study 2: PRE and POST) and condition (Study 1: HL vs. HV; Study 2 and HL vs. HV). *Post hoc* pairwise comparisons were made when interactions reached statistical significance to further investigate the simple main effects of time and/or condition. Note that Study 1 and Study 2 participant numbers were established based on prior study designs implementing within‐leg condition designs (Mitchell et al., 2012; Morton et al., 2019). As the current study represents a secondary analysis of previously collected samples, *post hoc* power analyses were not conducted. While we acknowledge that the modest sample sizes (Study 1 *n* = 11 and Study 2 *n* = 12–15) may have limited our ability to detect smaller between‐condition effects, we believe that the strength of these analyses was that both studies employed within‐subject/between‐leg designs which significantly reduces variability compared to parallel group designs (MacInnis et al., 2017).

## 3 RESULTS

### 3.1 Study 1: Effects of acute bouts of HV versus HL on markers of mitochondrial biogenesis

**FIGURE 2:**
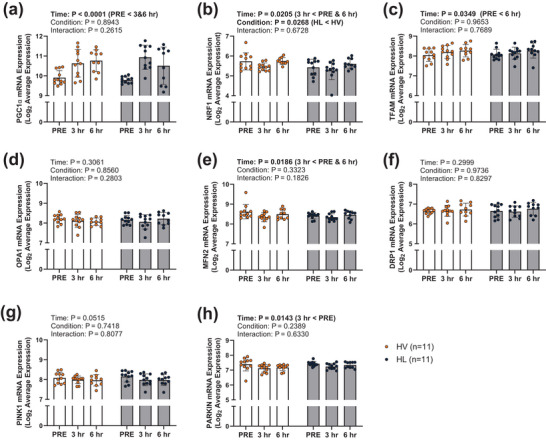
Study 1 mRNA expression data. mRNA expression from Study 1 participants (with *n* = 11 at PRE and 3 h and *n* = 10 at 6 h) reported as log_2_ average expression (provided by mRNA array analysis). Bar graphs contain mean and standard deviation bars, with individual respondent values overlaid as individual datapoints. Orange data points and dark grey bars represent the high‐volume resistance training while dark blue data points and light grey bars represent high load resistance training. Each condition includes PRE as well as 3‐ and 6‐h post‐exercise time points. DRP1, dynamin‐related protein 1; HL, high load; HV, high volume; MFN2, mitofusion 2; NRF1, nuclear respiratory factor 1; OPA1, optic atrophy 1; PGC1α, peroxisome proliferator‐activated receptor γ coactivator‐1 α; PINK1, PTEN induced putative kinase 1; TFAM, mitochondrial transcription factor A.

In the 11 Study 1 participants (with *n* = 11 at PRE and 3 h and *n* = 10 at 6 h), all markers of mitochondrial biogenesis demonstrated significant main effects of time, but no condition × time interactions were observed. Specifically, PGC1α mRNA increased from PRE to 3 h (*post hoc P* < 0.0001) and 6 h (*post hoc P* = 0.0004) (Figure 2a), NRF1 mRNA expression decreased from PRE to 3 h (*post hoc P* = 0.0239) but returned to baseline at 6 h (PRE vs. 6‐h *post hoc P* = 0.6293, Figure 2b), and TFAM mRNA expression increased from PRE to 6 h (*post hoc P* = 0.0281, Figure 2c). Among all markers of mitochondrial remodelling, only two markers demonstrated main effects of time with no main effects of RT condition or condition × time interactions. MFN2 mRNA expression decreased from PRE to 3 h (*post hoc P* = 0.0110) but returned to baseline by 6 h (PRE vs. 6‐h *post hoc P* = 0.4545, Figure 2e) and Parkin mRNA expression decreased from PRE to 3 h (*post hoc P* = 0.0033, Figure 2h).

### 3.2 Study 2: General training characteristics and adaptations to chronic RT training

General training characteristics and adaptations (body composition, VL thickness and pennation angle, leg extensor peak torque and three repetition maximum testing, and maximal strength testing) of all 15 Study 2 participants were previously described by Vann and colleagues, and the reader is encouraged to refer to Vann et al. (2022). In summary, a significant interaction was evident for vastus lateralis muscle cross‐sectional area (assessed via magnetic resonance imaging; interaction *P* = 0.0460), where HV increased this metric from PRE to POST (+3.2%, *P* = 0.0180), but HL training did not (−0.1%, *P* = 0.4750). Interestingly, 6‐week integrated non‐myofibrillar protein synthesis rates were also higher in the HV versus HL leg (*P* = 0.0180), while no difference between legs existed for integrated myofibrillar protein synthesis rates (*P* = 0.6870).

### 3.3 Study 2: Effect of chronic HV versus HL RT on mRNA expression on markers of mitochondrial biogenesis and remodelling

**FIGURE 3:**
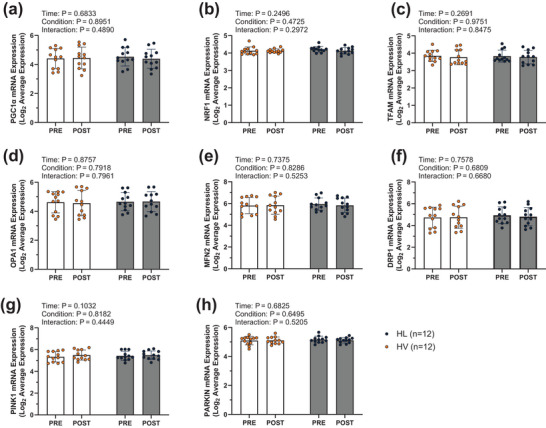
Study 2 mRNA expression data. mRNA expression from Study 2 participants (*n* = 12) reported as log_2_ average expression (provided by mRNA array analysis). Bar graphs contain mean and standard deviation bars, with individual respondent values overlaid as individual datapoints. Orange data points and dark grey bars represent the high‐volume resistance training leg while dark blue data points and light grey bars represent high load resistance training leg (within‐subject). Each condition includes PRE and POST intervention time points. DRP1, dynamin‐related protein 1; HL, high load; HV, high volume; MFN2, mitofusion 2; NRF1, nuclear respiratory factor 1; OPA1, optic atrophy 1; PGC1α, peroxisome proliferator‐activated receptor γ coactivator‐1 α; PINK1, PTEN induced putative kinase 1; TFAM, mitochondrial transcription factor A.

In 12 of the 15 Study 2 participants that had available mRNA data, 6 weeks of chronic RT did not affect the mRNA expression of genes related to mitochondrial biogenesis and remodelling (Figure 3). Specifically, no significant main effects of time or interactions were evident for PGC1α mRNA (Figure 3a), NRF1 mRNA (Figure 3b), TFAM mRNA (Figure 3c), OPA1 mRNA (Figure 3d), MFN2 mRNA (Figure 3e), DRP1 mRNA (Figure 3f), PINK1 mRNA (Figure 3g), or Parkin mRNA (Figure 3h); all main effects and interaction *P*‐values are show in the figure.

### 3.4 Study 2: Effects of chronic HV versus HL RT on mitochondrial biogenesis and remodelling proteins as well as CS activity

**FIGURE 4:**
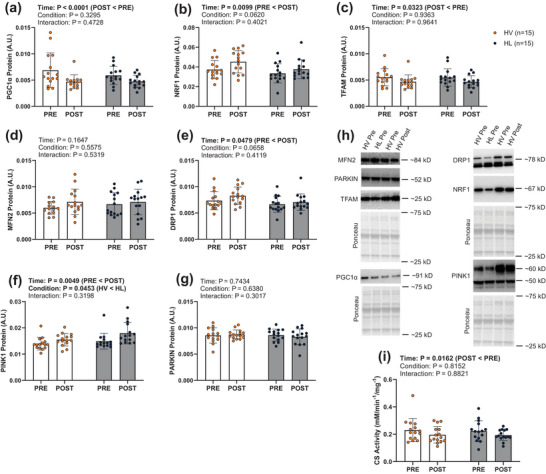
Study 2 mitochondrial proteins and CS activity data. Targeted mitochondrial protein levels and CS activity levels from Study 2 participants (*n* = 15). (a–g) Bar graphs contain mean and standard deviation bars, with individual respondent values overlaid as individual datapoints. Orange data points and dark grey bars represent the high‐volume resistance training leg while dark blue data points and light grey bars represent high load resistance training leg (within‐subject). Each condition includes PRE and POST intervention time points. (h) Each protein is displayed on the representative western blot image. CS, citrate synthase; DRP1, dynamin‐related protein 1; HL, high load; HV, high volume; MFN2, mitofusion 2; NRF1, nuclear respiratory factor 1; PGC1α, peroxisome proliferator‐activated receptor γ coactivator‐1 α; PINK1, PTEN induced putative kinase 1; TFAM, mitochondrial transcription factor A.

Interrogation of proteins associated with mitochondrial biogenesis in response to chronic HV and HL RT revealed only main effects of time where PGC1α (*P* < 0.0001, Figure 4a) and TFAM (*P* = 0.0323, Figure 4c) protein expression decreased from PRE to POST while NRF1 protein expression increased from PRE to POST (*P* = 0.0099, Figure 4b). However, there were no significant condition × time interactions observed for these three markers. Among markers of mitochondrial remodelling in response to chronic bouts of HV and HL RT, DRP1 and PINK1 demonstrated main effects of time with protein expression higher at POST for both markers (*P* = 0.0479 and *P* = 0.0049, respectively, Figure 4e,f). However, there were no significant condition × time interactions observed. CS activity in response to HV and HL RT demonstrated a main effect of time where CS activity decreased from PRE to POST (*P* = 0.0162, Figure 4i). However, no significant condition × time interaction was observed.

## 4 DISCUSSION

The purpose of this study was to investigate the acute transcriptional and chronic translational responses of mitochondrial specific genes/proteins to HL versus HV RT. Specifically, we accomplished this by interrogating previously collected data from two separate groups of participants who had completed either acute bouts of HV and HL RT (Study 1) or completed a chronic RT programme also including HV and HL RT (Study 2). Based on previous findings, we hypothesized that HV training would have a more robust effect on mitochondrial markers than HL training; however, given the findings of the present study, this hypothesis was not supported.

Our primary finding is that RT can elicit alterations to markers of mitochondrial biogenesis and remodelling regardless of RT paradigm. We posit that for such populations it is possible that participating in RT could provide some benefit to mitochondrial adaptations in addition to more characterized benefits such as increased lean mass and strength. To this end, investigations into RT's effects on mitochondrial adaptations have reported similar benefits. A RT study from Burd et al. (2012) interrogating the effects of an acute bout of RT on mitochondrial protein synthesis rates and PGC1α mRNA expression demonstrated increases in mitochondrial protein content at 6‐ and 24‐h post‐RT bout and a 3‐fold increase in PGC1α mRNA expression 6‐h post‐RT bout. However, it is important to note that reports on the effect of chronic RT on mitochondrial markers vary (Groennebaek & Vissing, 2017; Parry et al., 2020). Here we report a decrease in CS activity following RT, suggesting that mitochondrial volume density decreased. However, Tang et al. quantified CS activity before and after a 12‐week RT protocol and reported an increase in this outcome (Tang et al., 2006). Readers should appreciate that, as muscle hypertrophy ensues with RT, no change in tissue CS activity levels likely indicates that mitochondrial volume density proportionally increases with cell growth. Conversely, if muscle CS activity levels decrease with RT, this may reflect a lag in the expansion of mitochondrial volume density relative to cell growth (Parry et al., 2020). In this regard, a comprehensive review by Groennebaek & Vissing (2017) cites numerous RT studies reporting that no change or a decrease in muscle CS activity levels occurs, and we have similarly shown that CS activity levels decrease in trained and untrained participants following periods of RT (Haun et al., 2019; Roberts et al., 2018). These collective reports have led us to speculate that changes in muscle CS activity with RT are likely dependent upon the training status and age of the participant, where increases in this marker are typically observed in untrained subjects (especially older untrained participants) (Parry et al., 2020). Recent data from our laboratory also add additional insight into this area. We assessed CS activity changes following 10 weeks of RT in untrained participants and compared these data to what was observed via histology using a highly specific mitochondrial antibody (TOMM20) (Ruple et al., 2021). Our histological analysis indicated mitochondrial volume density significantly increased, and in fact, outpaced muscle fibre growth, whereas tissue CS activity levels were not altered. Moreover, no association was evident when examining the pre‐to‐post intervention change scores in both metrics. Given these recent data, CS activity assay may lack the sensitivity to track changes in mitochondrial volume density with RT interventions, especially in studies involving previously trained participants where adaptations are likely less robust. In line with this hypothesis, the current data suggest that mitochondrial volume density is marginally affected with either form of training, and this may have been due to training status of the participants.

We also observed that the mRNA response to an acute bout of resistance exercise was not always indicative of translational response to chronic resistance exercise. For instance, though Study 1 results indicate PGC1α mRNA expression increases 3 and 6 h following an acute bout of HV or HL RT, Study 2 indicates chronic RT did not result in a complimentary increase in PGC1α mRNA or protein expression. In fact, PGC1α protein expression was decreased following chronic RT. Parkin and MFN2 also demonstrated changes in mRNA expression following an acute bout of resistance exercise; however, neither gene demonstrated any change in mRNA or protein expression following chronic resistance exercise. Conversely, neither DRP1 nor PINK1 showed any transcriptional response to an acute bout of resistance exercise, though chronic RT resulted in statistically significant changes in protein expression for these genes. While these data suggest a disconnect between the transcriptional response to acute training versus the translational response with chronic training, it is critical to note that these data were collected from different participants undergoing different training paradigms. Therefore, these data are difficult to compare. Participant and protocol differences notwithstanding, these findings and the argument presented by Miller and colleagues reiterate the notion that mRNA expression alone is not representative of long term adaptations (Miller et al., 2016). While this may appear to be yet another challenge in the accurate determination of molecular signalling events following RT, this disconnect may shed light on key mechanisms of molecular modulation in RT adaptation. Considering the divergence observed between the initial transcriptional response and subsequent translational response of certain genes, we postulate that additional factors affecting the molecular response to resistance exercise may be at play. It is possible that epigenetic modifications (e.g. micro RNAs, long non‐coding RNAs, DNA methylation, histone modifications) play a role in the protein expression of such transcribed mitochondrial mRNAs (Egan & Sharples, 2023). Given these discordant findings and the established RT responsiveness of epigenetic factors, investigations into the epigenetic modifications that might impact the protein expression (or lack thereof) of transcribed mitochondrial mRNAs in response to RT is warranted.

### 4.1 Limitations

One limitation was our participant population. The original intent of both studies was to examine responses in trained men, who are typically more motivated to maximize muscle hypertrophy with RT, whereas women self‐report other motivations (e.g., achieving muscle toning and body mass management) (Nuzzo, 2023). Considering that only trained, male participants were utilized in this investigation, these results cannot be generalized to female or untrained populations. Another limitation was that the molecular focus of this study was not accompanied by functional measures (e.g., ${\dot{V}}_{O_{2}\max}$ changes) that indicate changes within the whole system. The short training duration of Study 2 is a limitation. It is possible that 6 weeks might not have provided sufficient stimulus to observe differences between each training condition in previously trained participants. Likewise, although the HV leg in Study 2 did experience significantly more volume as we have previously reported (Vann et al., 2022), this was only an 11% difference and this lack of appreciable training volume differences between legs may have been a chief reason as to why we observed several null findings. In this regard, longer training studies that measure these markers with more pronounced volume differences between legs are needed to determine how the muscle‐metabolic milieu adapts in response to different RT paradigms. Another limitation is the lack of a time‐only control group in both studies. Indeed, we attempted to examine if the Metamex online data repository possessed transcriptomic data from acute or chronic non‐exercise trials for comparative purposes to Study 1 outcomes (Pillon et al., 2020). However, Metamex only possesses study designs like ours whereby acute post‐exercise responses are examined within‐subject without considering a non‐exercise control limb. Moreover, had a non‐exercise control group been implemented in Study2, then any within‐group effects would have likely been more attributable to a deconditioning effect given that participants had prior RT. Thus, while we recognize that including non‐exercise control conditions in both studies may have provided additional information, we also posit that resultant data would have presented additional limitations (particularly in Study 2). Finally, it should be noted that, while we chose to adopt a significance threshold of *P *< 0.05 throughout, the number of analyses conducted presents an increased possibility for a type I error.

### 4.2 Conclusion

Although RT resulted in both acute transcriptional (mRNA) and chronic CS activity and translational (protein) responses, there was little effect of RT paradigm (HV or HL) in markers of mitochondrial biogenesis and remodelling. These results indicate that RT can alter mitochondrial markers both acutely and chronically regardless of HV or HL RT mode. Additionally, we observed that chronic protein level responses did not necessarily reflect transcriptional responses, though these data were collected in a separate cohort of participants. Nonetheless, these results point to possible epigenetic modifications to the molecular events following acute and chronic resistance exercise. Therefore, additional measures besides mRNA and protein expression must be taken to comprehensively assess molecular events following resistance exercise.

## AUTHOR CONTRIBUTIONS

Breanna J. Mueller, Carlton D. Fox, Michael D. Roberts, and Andreas N. Kavazis: conceptualization, analysis, and writing of the primary draft. Casey L. Sexton, Christopher G. Vann, and Michael D. Roberts: experimentation. Hailey A. Parry, Paulo H. C. Mesquita, Bradley A. Ruple, Joshua S. Godwin, Mason McIntosh, Darren T. Beck, Kaelin C. Young, and Stuart M. Philips: collaborative insight throughout experiments and/or significant resources devoted to project outcomes; all‐co‐authors: manuscript editing and approval of final submission. All authors have read and approved the final version of this manuscript and agree to be accountable for all aspects of the work in ensuring that questions related to the accuracy or integrity of any part of the work are appropriately investigated and resolved. All persons designated as authors qualify for authorship, and all those who qualify for authorship are listed.

## CONFLICT OF INTEREST

None of the authors have conflicts of interest in relation to the current data. In the interest of full transparency, the laboratory of M.D.R. does perform contracted studies from industry various sponsors. M.D.R. has served as a paid consultant to various industry entities in line with Auburn University's Conflict of Interest Policies. S.M.P. has received grant funding from the Canadian Institutes of Health Research, the National Science and Engineering Research Council of Canada, the US National Institutes of Health, Roquette Frères, Nestlé Health Sciences, FrieslandCampina, the US National Dairy Council, Dairy Farmers of Canada, Myos, and Cargill. S.M.P. has received travel expenses and honoraria for speaking from Nestlé Health Sciences, Optimum Nutrition, Nutricia, and Danone. S.M.P. holds patents licensed to Exerkine Inc. but reports no financial gains from these patents or otherwise.

## Data Availability

All individual respondent data can be visualized in figures. Raw data will be made available by the corresponding author (mdr0024@auburn.edu) upon reasonable request.
